# Super fragmented: a nationally representative cross-sectional study exploring the fragmentation of inpatient care among super-utilizers

**DOI:** 10.1186/s12913-021-06323-5

**Published:** 2021-04-14

**Authors:** Zach Kaltenborn, Koushik Paul, Jonathan D Kirsch, Michael Aylward, Elizabeth A. Rogers, Michael T. Rhodes, Michael G. Usher

**Affiliations:** 1 Department of Medicine, Division of General Internal Medicine, University of Minnesota Medical School, 420 Delaware St. SE MMC 741, Minneapolis, MN 55455 USA; 2 Department of Pediatrics, University of Minnesota Medical School, Minneapolis, MN 55455 USA

**Keywords:** Hospital super-utilizer, Fragmentation, Socioeconomic health disparities

## Abstract

**Background:**

Super-utilizers with 4 or more admissions per year frequently receive low-quality care and disproportionately contribute to healthcare costs. Inpatient care fragmentation (admission to multiple different hospitals) in this population has not been well described.

**Objective:**

To determine the prevalence of super-utilizers who receive fragmented care across different hospitals and to describe associated risks, costs, and health outcomes.

**Research design:**

We analyzed inpatient data from the Health Care Utilization Project’s State Inpatient and Emergency Department database from 6 states from 2013. After identifying hospital super-utilizers, we stratified by the number of different hospitals visited in a 1-year period. We determined how patient demographics, costs, and outcomes varied by degree of fragmentation. We then examined how fragmentation would influence a hospital’s ability to identify super-utilizers.

**Subjects:**

Adult patients with 4 or more inpatient stays in 1 year.

**Measures:**

Patient demographics, cost, 1-year hospital reported mortality, and probability that a single hospital could correctly identify a patient as a super-utilizer.

**Results:**

Of the 167,515 hospital super-utilizers, 97,404 (58.1%) visited more than 1 hospital in a 1-year period. Fragmentation was more likely among younger, non-white, low-income, under-insured patients, in population-dense areas. Patients with fragmentation were more likely to be admitted for chronic disease management, psychiatric illness, and substance abuse. Inpatient fragmentation was associated with higher yearly costs and lower likelihood of being identified as a super-utilizer.

**Conclusions:**

Inpatient care fragmentation is common among super-utilizers, disproportionately affects vulnerable populations. It is associated with high yearly costs and a decreased probability of correctly identifying super-utilizers.

**Supplementary Information:**

The online version contains supplementary material available at 10.1186/s12913-021-06323-5.

## Introduction

Care fragmentation is defined as the dispersion of an individual’s health care across systems and providers and is ubiquitous in the U.S. healthcare system [1]. Fragmentation is a major contributor to low-quality care, negative health outcomes, and high costs [2–4]. Previous studies show that fragmentation is common in the outpatient setting with nearly one third of Medicare beneficiaries transitioning into a different health system in a 1-year period [5]. Similarly, patients with commercial insurance and chronic illnesses such as cardiovascular disease and diabetes receive lower-quality care, incur higher costs, and have higher rates of preventable hospitalizations when their care is fragmented [6].

Reducing unnecessary hospitalizations is an important area of focus to provide higher-value care. Patients with a high degree of hospital use, termed super-utilizers, are admitted 4 or more times over a 1-year period, incur higher costs, and receive lower overall care quality [7]. Within the Medicaid population, 5% of beneficiaries accumulate upwards of 50% of total expenditures, with acute care utilization driving much of the spending [8]. In addition to having a high prevalence of multiple chronic medical conditions, mental health diagnoses and substance abuse, super-utilizers also disproportionately tend to be from racial minority groups, and have a large burden of unmet social needs [9–13]. Targeted interventions, such as high-intensity outpatient care, have been enacted to address the needs of super-utilizers with mixed efficacy [14–17].

One unaddressed factor that could contribute to lower-quality care, risk of readmission, and higher rates of utilization within this population is fragmentation of inpatient care. Our understanding of this problem is limited because the majority of studies of fragmentation focus on readmissions to other hospitals over a short period of time [18]. Two studies have begun to explore this group using single-state or urban regional data, and they demonstrate that as many as 20% of hospital super-utilizers experience some degree of fragmentation [19, 20]. A better understanding of inpatient care fragmentation is needed to identify its impact on patient outcomes as well as to design interventions that reduce gaps in care quality and reduce costs. For example, inpatient care fragmentation could mask an individual patient’s super-utilizer status particularly in the absence of uniform inter-operability of electronic health records. Additionally, when care is received at disparate centers, effective care coordination becomes more difficult. To explore the impact of inpatient care fragmentation we aimed to establish its prevalence within a multi-state sample of hospital super-utilizers. We then describe the features of patients with highly fragmented inpatient care, illustrate its potential impact on outcomes and cost, and compare strategies that may aid in identifying this vulnerable population.

## Methods

### Database development and patient selection

We created a large administrative dataset for cross-sectional analysis of the Health Care Utilization Project’s (HCUP) State Inpatient and Emergency Department Database for six states in 2013 (VT, NY, FL, IA, GA, and UT) [21]. These data account for nearly 97% of each state’s emergency department (ED) and inpatient encounters during the study period. We selected these states based on the presence of a unique patient identifier (VisitLink ID) in both the inpatient and ED datasets which allowed for capturing utilization across settings. We relied on a single year, to prevent oversampling across multiple years. This study was approved by the University of Minnesota Institutional Review Board (Study #00005624) and was conducted in accordance with both HCUP’s data use agreement and internal standards.

Patients over the age of 18 were identified by VisitLink ID, sex, and age. We only included hospital super-utilizers, defined as four or more inpatient encounters in a 1-year period [7]. To ensure the robustness of our findings, we repeated all statistical analyses using the top 95th percentile of inpatient costs for each state as another method of identifying super-utilizers. We excluded all scheduled inpatient encounters, admissions for rehabilitation, and admissions for childbirth and labor. Inter-hospital transfers were considered two separate admissions. To ensure that did not substantially bias our findings, we investigated the impact of compressing inter-hospital transfers and same-day readmissions into one admission both for purposes of identifying super-utilizers as well as fragmentation.

### Outcomes and measures

Fragmentation was measured as the number of different acute hospitals to which an individual patient was admitted over a 1-year period. In this case, we define inpatient encounter as an unscheduled inpatient admission. We extracted data for patients’ age, sex, race and primary payer. Missing data for race and insurance were categorized as “other.” Since primary payer could change over the year, we present the per-encounter average as opposed to per-individual. Patients were categorized by income quartile and population density of the patient’s home location. Because housing instability and the process of losing or regaining insurance coverage might affect care fragmentation, we classified patients as either uninsured or homeless if that was coded at least once during the year. Lastly, we compared fragmentation groups for the ten most common admitting diagnoses and the presence of comorbid chronic conditions defined by the Agency for Healthcare Research and Quality (AHRQ) [22]. In addition to comparing individual comorbidities, we also clustered patients by presence of three or more chronic medical comorbidities, psychiatric illness, or substance abuse.

Outcome variables included measures of healthcare utilization in terms of number of admissions, length of stay, ED encounters and costs. We estimated cost by normalizing total charges to all-payer cost-to-charge ratio (CCR). If hospital-specific CCR was not available, we used the weighted group average cost-to-charge ratio [23]. We report demographic data for each group as a number and percentage of a dichotomous variable, mean and standard deviation for normally distributed continuous variables, and median and Interquartile Range (IQR) for skewed variables.

### Statistical analyses

We took three statistical approaches to better understand fragmentation among super-utilizers. First, we identified how patient factors, costs, and diagnoses, varied among patients with different degrees of inpatient fragmentation. We used chi-square and one-way ANOVA where appropriate, adjusting for repeated measures using Bonferroni.

Next, to determine whether specific diagnoses were associated with a greater degree of fragmentation, we performed a multivariate Poisson regression with the number of different hospitals encounters per year as the dependent variable while adjusting for patient age, race, population density and number of total inpatient encounters.

Finally, we conducted a final analysis to assess the ability of any single hospital to identify super-utilizers with and without inpatient care fragmentation. We first determined each individual hospital’s threshold for super-utilization as defined by several approaches to detecting super utilizers: top 5% of inpatient days, top 5% of yearly cost, and more than three or more than four inpatient admissions in one year (at a single hospital) [24–27]. We then calculated the sensitivity of each approach to identify super-utilizers (identified from multihospital data) who would have met any of these thresholds at a particular hospital. We compared the sensitivity, specificity, and C-statistic of each approach to identify super-utilizers, stratified by whether they visited one or more than one hospital.

## Results

We identified 167,515 super-utilizers with four or more inpatient visits in a 1-year period out of an initial cohort of 3,619,297 patients. Of these, 97,404 (58.1%) were admitted to more than 1 hospital, and 34,165 patients (20.3%) were admitted to three or more different hospitals in that year. Patient characteristics by group are summarized in Table 1. Overall, patients with higher inpatient fragmentation were younger and more likely to be non-white, on Medicaid or uninsured, and living in population-dense areas in more impoverished neighborhoods; they were also more likely to be denoted uninsured or homeless at least once during the year. All of these associations were dose-dependent, such that the greater the fragmentation, the more likely patients were to have been homeless, uninsured, or from urban areas with a lower income bracket.

**Table 1:** Characteristics of super-utilizers stratified by the number of hospitals encountered in 1 year

Number of Different Hospitals	1	2	3	4	5	6	7	8	p
N (%)	70,111	63,239	24,527	6615	1812	702	347	162	
Age, years (%)	64.73 (18.18)	62.61 (17.9)	58.81 (17.94)	53.41 (17.08)	49.3 (14.93)	46.2 (12.53)	45.46 (11.78)	45.55 (12.84)	< 0.001
Female, n (%)	37,299 (53.2%)	32,115 (50.78%)	11,672 (47.59%)	2754 (41.63%)	612 (33.77%)	210 (29.91%)	82 (23.63%)	32 (19.75%)	< 0.001
White, n (%)	42,167 (60.14%)	34,989 (55.33%)	11,782 (48.04%)	2621 (39.62%)	603 (33.28%)	194 (27.64%)	76 (21.9%)	28 (17.28%)	< 0.001
Black, n (%)	14,083 (20.09%)	12,922 (20.43%)	5502 (22.43%)	1646 (24.88%)	475 (26.21%)	176 (25.07%)	92 (26.51%)	49 (30.25%)	< 0.001
Hispanic, n (%)	8155 (11.63%)	8037 (12.71%)	3565 (14.54%)	1084 (16.39%)	311 (17.16%)	131 (18.66%)	60 (17.29%)	22 (13.58%)	< 0.001
Other/Missing, n (%)	5706 (8.14%)	7291 (11.53%)	3678 (15%)	1264 (19.11%)	423 (23.34%)	201 (28.63%)	119 (34.29%)	63 (38.89%)	< 0.001
Medicare, n (%)	45,129 (64.37%)	38,955 (61.6%)	13,855 (56.49%)	3061 (46.27%)	742 (40.93%)	211 (30.06%)	94 (27.05%)	45 (27.92%)	< 0.001
Medicaid, n (%)	11,159 (15.92%)	11,545 (18.26%)	5736 (23.38%)	2173 (32.86%)	759 (41.88%)	368 (52.39%)	201 (57.93%)	94 (57.77%)	< 0.001
Private, n (%)	9283 (13.24%)	8417 (13.31%)	3058 (12.47%)	697 (10.53%)	143 (7.89%)	48 (6.81%)	17 (5.02%)	6 (3.54%)	< 0.001
Uninsured, n(%)	2186 (3.12%)	2197 (3.47%)	1044 (4.26%)	418 (6.32%)	107 (5.92%)	48 (6.77%)	25 (7.12%)	10 (6.26%)	< 0.001
Other/Missing, n(%)	2354 (3.4%)	2125 (3.4%)	834 (3.4%)	266 (4.0%)	61 (3.4%)	27 (3.8%)	10 (2.9%)	7 (4.3%)	0.028
Zip Income Quartile 1 n (%)	23,007 (32.82%)	20,164 (31.89%)	7328 (29.88%)	1818 (27.48%)	464 (25.61%)	148 (21.08%)	73 (21.04%)	34 (20.99%)	< 0.001
Zip Income Quartile 2 n (%)	18,899 (26.96%)	17,769 (28.1%)	6800 (27.72%)	1721 (26.02%)	433 (23.9%)	158 (22.51%)	59 (17%)	40 (24.69%)	< 0.001
Zip Income Quartile 4 n (%)	15,048 (21.46%)	13,949 (22.06%)	5759 (23.48%)	1656 (25.03%)	449 (24.78%)	174 (24.79%)	81 (23.34%)	33 (20.37%)	< 0.001
Zip Income Quartile 4 n (%)	11,495 (16.4%)	10,679 (16.89%)	4503 (18.36%)	1397 (21.12%)	461 (25.44%)	221 (31.48%)	134 (38.62%)	55 (33.95%)	< 0.001
Large Metropolitan (> 1 million), n (%)	41,252 (58.84%)	38,503 (60.88%)	16,102 (65.65%)	4703 (71.1%)	1376 (75.94%)	559 (79.63%)	287 (82.71%)	130 (80.25%)	< 0.001
Small Metropolitan (< 1 million), n (%)	21,070 (30.05%)	16,363 (25.87%)	5091 (20.76%)	1134 (17.14%)	265 (14.62%)	97 (13.82%)	41 (11.82%)	27 (16.67%)	< 0.001
Micropolitan, n (%)	4870 (6.95%)	4889 (7.73%)	1803 (7.35%)	403 (6.09%)	92 (5.08%)	28 (3.99%)	12 (3.46%)	3 (1.85%)	< 0.001
Rural, n (%)	2793 (3.98%)	3438 (5.44%)	1522 (6.21%)	371 (5.61%)	78 (4.3%)	18 (2.56%)	7 (2.02%)	2 (1.23%)	< 0.001
Ever Uninsured n (%)	3707 (5.29%)	4457 (7.05%)	2319 (9.45%)	948 (14.33%)	295 (16.28%)	134 (19.09%)	73 (21.04%)	43 (26.54%)	< 0.001
Ever Homeless n (%)	1311 (1.87%)	2843 (4.5%)	2237 (9.12%)	1210 (18.29%)	553 (30.52%)	297 (42.31%)	192 (55.33%)	102 (62.96%)	< 0.001

Inpatient hospital utilization is summarized in Table 2. Patients with a higher degree of fragmentation were more likely to be discharged home without home health care or to transitional care and more likely to leave against medical advice. A higher rate of fragmentation was associated with lower hospital-reported mortality. Readmissions to other hospitals were more common with higher degrees of fragmentation. However, readmissions to other hospitals accounted for only 19.9% of encounters in patients with any degree fragmentation. Patients with higher rates of fragmentation were associated with higher yearly costs. The increase in cost was driven by higher admission rates (Supplemental Figure 2) as per-encounter cost and length of stay were actually lower among patients with higher fragmentation (Table 2). Patients also differed by admitting diagnoses. Super-utilizers who visited one or two hospitals were most commonly admitted for acute illness and acute exacerbations of chronic disease. In contrast, patients visiting three or more hospitals were most likely to be admitted for management of chronic illness, psychiatric disease, or substance abuse (Table 3).

**Table 2:** Yearly hospital utilization stratified by the number of hospitals a patient encountered in a 1-year period

Number of Different Hospitals		1	2	3	4	5	6	7	8	p
Yearly Totals	Inpatient Encounters (median, IQR)	4 (4–5)	5 (4–6)	5 (4–6)	6 (5–8)	8 (6–11)	10 (8–14)	13 (10–17)	15 (12–19)	< 0.001
	Inpatient Days (median, IQR)	25 (17–38)	26 (17–41)	30 (19–49)	37 (22–59)	47 (29–77)	56 (36–84)	64 (42–102)	77 (48–112)	< 0.001
	Inpatient Cost in thousands of US dollars, (median, IQR)	51 (31–90)	55 (33–99)	60 (34–113)	61 (34–118)	69 (39–129)	72 (44–117)	75 (50–133)	93 (60–138)	< 0.001
	Unadjusted Mortality, n (%)	6253 (8.92)	5660 (8.95)	1931 (7.87)	387 (5.85)	73 (4.03)	14 (1.99)	8 (2.31)	4 (2.47)	< 0.001
Per Encounter	Length of Stay, mean (SD)	6.24 (7.33)	6.46 (8.01)	6.72 (8.77)	6.62 (8.81)	6.25 (8.18)	5.72 (7.17)	5.29 (6.52)	5.4 (6.48)	< 0.001
	Cost in thousands of US dollars, mean (SD)	12 (18)	13 (19)	12 (19)	10 (15)	8 (13)	7 (11)	7 (9)	7 (9)	< 0.001
	30 Day Readmission, n (%)	143,682 (41.3%)	134,419 (40.9%)	59,692 (42.4%)	22,668 (48.9%)	9902 (58.0%)	5284 (64.3%)	3528 (70.5%)	1823 (70.2%)	< 0.001
	30 Day Readmission to Same Hospital, n (%)^a^	143,682 (100%)	89,049 (66.3%)	26,753 (44.8%)	7478 (33.0%)	2634 (26.6%)	1112 (21.0%)	650 (18.4%)	224 (12.3%)	< 0.001
	30 Day Readmissions to another Hospital, n(%)^a^	0 (0%)	45,370 (33.8%)	32,939 (55.2%)	15,190 (67.0%)	7268 (73.4%)	4172 (79.0%)	2878 (81.6%)	1559 (85.5%)	< 0.001
	Discharged to skilled nursing facility, n (%)^b^	75,197 (22.0%)	72,361 (22.4%)	30,012 (21.6%)	8602 (18.7%)	2661 (15.6%)	1027 (12.5%)	549 (11.0%)	229 (8.9%)	< 0.001
	Discharged Home, n (%)^b^	180,623 (51.9%)	166,927 (50.6%)	74,505 (53.7%)	27,400 (59.6%)	10,919 (64.2%)	5593 (68.2%)	3380 (67.6%)	1815 (70.1%)	< 0.001
	Discharged with Home Health^c^, n (%)^b^	77,079 (22.6%)	60,471 (18.7%)	20,074 (14.5%)	4437 (9.7%)	1068 (6.9%)	296 (3.6%)	114 (2.9%)	49 (1.9%)	< 0.001
	Transferred to Other Hospital, n (%)^b^	3224 (0.9%)	14,470 (4.5%)	7920 (5.7%)	2225 (4.8%)	645 (3.8%)	188 (2.3%)	102 (2.0%)	66 (2.6%)	< 0.001
	Left AMA^d^, n (%)^b^	5526 (1.6%)	8997 (2.8%)	6217 (4.5%)	3336 (7.3%)	1720 (10.1%)	1098 (13.4%)	855 (17.1%)	432 (16.7%)	< 0.001

^a^ Percentage is a proportion of total 30-day readmissions

^b^ Percentage is a proportion of hospital survivors

^c^ Home Health: Includes patients discharged with home health services such as physical therapy or IV antibiotics

^d^: *AMA*:Against Medical Advice

**Table 3:**
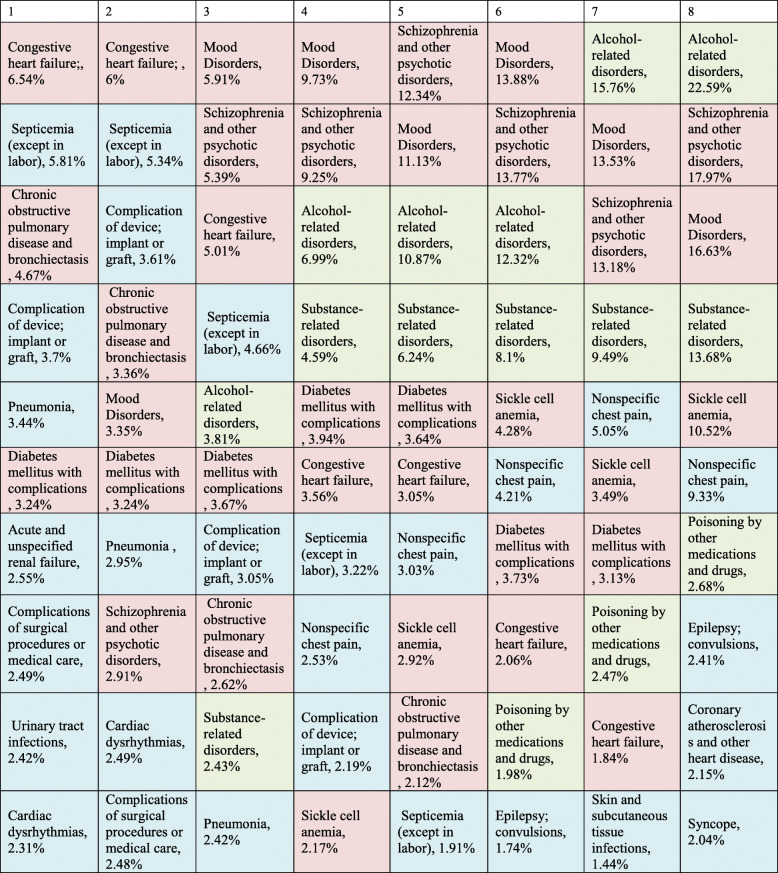
Differences in admitting diagnosis stratified by degree of inpatient fragmentation. Top ten most common admitting diagnoses per encounter. Blue (acute illness), Red (acute exacerbation of chronic illness, including psychiatric disease), Green (Substance use disorders)

We performed a multivariate analysis to determine what degree fragmentation related to chronic comorbidities when adjusting for patient demographics and number of encounters. Psychosis, drug and alcohol abuse, neurological disorders, obesity, and acquired immunodeficiency syndrome (AIDS). Conversely, lymphoma, metastatic cancer, renal failure or congestive heart failure (CHF) were associated with a lower degree of fragmentation (Supplemental Digital Content). Similarly, a greater degree of fragmentation was associated with lower rates of multiple medical comorbidities, and significantly higher rates of substance abuse and psychiatric illness (Supplemental Digital Content).

We investigated how inter-hospital transfers affected the rate of hospital fragmentation. While inter-hospital transfer rates were higher on average among patients with inpatient fragmentation, treating a transfer as a single hospital stay, as opposed to two separate stays reduced the measurement of fragmentation in only 2.9% of patients (Supplemental Digital Content). This was due to the fact that a majority of patients had additional encounters at both the referring and receiving hospital. Treating an inter-hospital transfer as a single stay at either sending or receiving hospital did not change any conclusion drawn in this study.

**Fig. 1:**
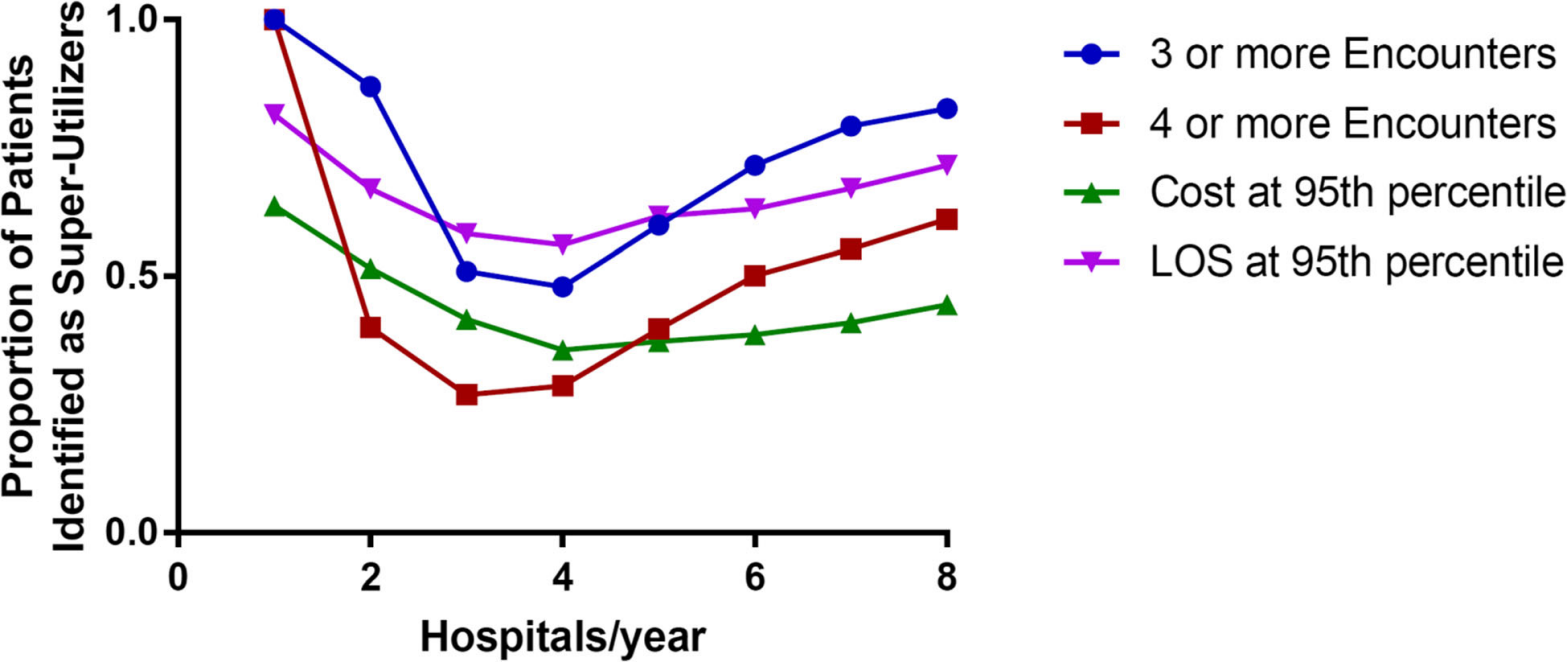
Proportion of patients identified as super-utilizers using single hospital data: Comparing 4 different single hospital methods to detect super-utilizers: 3 and 4 or more inpatient encounters, and top 5th percentile for either inpatient days and inpatient cost. We determined the likelihood that a single hospital would correctly identify a patient as a super-utilizer in the absence of data sharing

Finally, we compared four common methods of identifying super-utilizers from a single-hospital perspective: three or more inpatient stays, four or more inpatient stays, top 5% in yearly inpatient days, top 5% in yearly cost (Table 4). Relying solely on single-hospital data, using four or more hospitalizations (at a single hospital) as a threshold was 62.85% sensitive; this is compared to a sensitivity of 71.34% using top 5th percentile of yearly inpatient days; and 54.3% using top 5th percentile of yearly cost (Supplemental Digital Content). Sensitivity of an individual hospital’s ability to detect super-utilizers with fragmented care followed a U shape, with patients who visited 3,4, and 5 hospitals the least likely to be identified (Fig. 1). The ability of different measures to correctly identify super-utilizers when relying on single hospital data is outlined in Table 3. Overall performance of these measures showed fair to good prediction, but all measures performed more poorly among patients who visited multiple hospitals.

**Table 4:** Individual hospitals ability to accurately capture super-utilizers. C-statistics with 95% Confidence interval based on an individual patient’s maximum use at a single hospital

	One Hospital	2 or More Hospitals	Total
3 or more encounters	0.968 (0.967 to 0.968)	0.746 (0.745 to 0.749)	0.911 (0.910 to 0.912)
4 or more encounters	1.0 (Reference)	0.681 (0.679 to 0.682)	0.814 (0.813 to 0.815)
Length of Stay > 95 percentile	0.875 (0.874 to 0.877)	0.737 (0.735 to 0.738)	0.820 (0.819 to 0.821)
Yearly Cost > 95 percentile	0.788 (0.786 to 0.790)	0.667 (0.666 to 0.669)	0.738 (0.737 to 0.739)

Finally, given a mechanical relationship between number of encounters and number of hospitals, we repeated all analyses using an additional definition of super-utilizer: yearly cost above the 95th percentile for each state. Using this definition, associations between fragmentation and patient demographics, diagnoses, and detection rates were similar. Overall, yearly length of stay greater than the 95th percentile at an individual hospital showed the best prediction independent of super-utilizer definition and was the least dependent on degree of fragmentation.

## Discussion

In this cross-sectional multistate study, we assessed the prevalence and impact of inpatient care fragmentation among a group of inpatient super-utilizers. Over one half of super-utilizers in our sample experienced a degree of inpatient care fragmentation with over one fifth visiting 3 or more different hospitals in one year. In addition to having higher overall costs, patients with fragmented inpatient care were more likely to be non-white, uninsured or underinsured, and to have been homeless at some point during the year. They were also far less likely to be recognized as super-utilizers by multiple methods. We make several contributions to the literature.

First, while a majority of the study of inter-hospital fragmentation has focused on readmissions to other hospitals, we show that that is just the tip of the iceberg [18]. We find that a majority of readmissions to other hospitals occur in patients who use multiple hospitals beyond a 30-day window. Additionally, readmissions to the same hospital capture only a small percentage of total healthcare utilization among patients with a high degree of fragmentation. Thus, the impact of inter-hospital fragmentation is likely to be far broader than commonly reported.

Second, we highlight the high degree of heterogeneity in demographics, diagnostic make-up, and hospital use patterns of super-utilizers [28]. High utilization may be driven by disease severity and complexity for which high-intensity, hospital-based care may improve survival, such as in heart failure [29]. In other cases, high readmission rates may be driven by insufficient preventative care, psychiatric care, housing, or addiction treatment. Stratifying by the degree of fragmentation separated patients with differing diagnostic make-up warranting different subspecialty care needs such as behavioral health. Our data suggest that a one-size-fits-all super-utilizer program will not be effective in fully addressing the underlying drivers of unnecessary hospital use.

Third, our study shows that identifying super-utilizers with fragmented care is challenging for individual hospitals. A patient may not meet a given institution’s threshold for being a super-utilizer if their inpatient encounters are spread across multiple hospitals. Within a single hospital, the highest performing approach for identifying super-utilizers across varying degrees of fragmentation was by using the top 5th percentile of yearly length of stay. However, this still only had a sensitivity of 0.71.

High-quality care coordination is an important aspect of addressing costs associated with the complex and vulnerable patient populations that define super-utilizers [30]. The combination of multiple disparate admission diagnoses from chronic medical conditions to substance abuse, along with the information gap caused by the lack of hospital electronic health record interoperability, creates a unique challenge for individual health systems to both identify at-risk patients and develop solutions to improve the care of high-utilizing patients. Due to information loss, hospitals may de-emphasize outpatient behavioral health, addiction treatment, and complex medical care. Given the lower risk of death of this population, and higher rates of admissions related to chronic illness, patients with highly fragmented care may benefit to a greater degree from high-intensity outpatient care with supports tailored to individual needs.

Directly assessing gaps in information such as engaging patients about fragmentation, integrating health information exchange data into routine practice, coordinating with regional departments of health and payers, and establishing regional data sharing agreements are necessary steps in developing successful super-utilizer programs. Without this approach, the needs of this vulnerable population will not be fully realized, and socioeconomic and racial disparities in care will persist. Overcoming information gaps among patients with a high degree of fragmentation is one mechanism by which health information technology may reduce racial and socioeconomic health disparities.

Our study has several limitations. First, we cannot separate the care of the patient inherent to the patient’s disease from the effect of inter-hospital fragmentation. This is particularly important given the socioeconomic and psychiatric risk factors that exist in this population. Additionally, while our data is designed to capture the care a patient received in the hospital setting, it does not establish the degree of care coordination or fragmentation that occurs outside the hospital, nor can we follow patients beyond a single calendar year. As this study is based on a large administrative dataset, results are subject to unadjusted confounders. Moreover, while statistically significant associations between diagnoses and fragmentation were observed, they may not be clinically meaningful or observable outside large populations. To address these challenges, clinical studies focused on identifying causative factors and interventions to reduce fragmentation are needed. Finally, this study suggests that data sharing through regional health information exchanges would be beneficial in capturing inpatient care fragmentation; however, specifically characterizing the effect of health information exchanges on utilization of high utilizers remains an important future direction [31, 32].

## Conclusions

To conclude, this study takes an important step by establishing that there is a significant and under-recognized population of super-utilizers who experience inpatient care fragmentation. We observed a strong association between socioeconomic risk factors, diagnostic make-up, and degree of fragmentation. Fragmentation also created a barrier for individual hospitals to accurately identify this subset of the super-utilizer population. Addressing inpatient care fragmentation among super-utilizers has the potential to improve patient outcomes and overall health equity.

## Supplementary Information

**Additional file 1.** Supplemental Figures and Tables including sensitivity analyis excluding inter-hospital transfers and defining super-utilizers as the top 95% percentile in yearly inpatient cost.

## Data Availability

All data analyzed in this study are available publicly from HCUP’s central distributor: https://www.distributor.hcup-us.ahrq.gov/. Public distribution of the primary datasets is limited by the data use agreement.
