# Supplementary material for: Super fragmented: a nationally representative cross-sectional study exploring the fragmentation of inpatient care among super-utilizers
Source: BMC Health Serv Res. 2021 Apr 14;21:338. doi: 10.1186/s12913-021-06323-5 (PMC8045386; doi:10.1186/s12913-021-06323-5)
Supplement: Supplementary file 1 — Additional file 1. Supplemental Figures and Tables including sensitivity analyis excluding inter-hospital transfers and defining super-utilizers as the top 95% percentile in yearly inpatient cost. [file 12913_2021_6323_MOESM1_ESM.docx]

Super Fragmented: A Nationally Representative Cross-Sectional Study Exploring the Fragmentation of Inpatient Care Among Super-Utilizers

Zach Kaltenborn MD^1,2^

Koushik Paul BA^1^

Jonathan Kirsch MD^1^

Michael Aylward MD^1,2^

Elizabeth A. Rogers MD MAS^1,2^

Michael T. Rhodes MD^1,2^

Michael G. Usher MD PhD^1^

1. Department of Medicine, Division of General Internal Medicine, University of Minnesota Medical School, Minneapolis, MN 55455
2. Department of Pediatrics, University of Minnesota Medical School, Minneapolis, MN 55455

**Supplemental Digital Content:**

1. Supplemental Table 1: Relationship between Chronic Comorbidities and Care Fragmentation

2. Supplemental Figure 1: Distribution of number of encounters per year by fragmentation

3. Supplemental Figure 2: Distribution of fragmentation with and without inter-hospital transfers treated as a single hospital stay.

4. Supplemental Table 2a - f: Patient demographics and fragmentation by state

5. Supplemental Figure 3: Rates of chronic medical illness, substance use, and psychiatric use among super utilizers with fragmentation.

6. Supplemental Table 3: Sensitivity and specificity of measures to identify super utilizers from individual hospitals; Super-utilizers defined as 4 or more hospitals in a 1 year period.

7. Supplemental Table 4: Patient demographics and fragmentation for super-utilizers defined as the top 95th percentile of inpatient costs.

8. Supplemental Figure 4: Distribution of number of encounters per year by fragmentation of super-utilizers defined as the top 95^th^ percentile of inpatient costs.

9. Supplemental Figure 5: Comparing 4 different single hospital methods to detect super-utilizers: 3 and 4 or more inpatient encounters, and top 5th percentile for either inpatient days or inpatient cost.

9. Supplemental Table 5: C-statistic for hospital level identification of super-utilizers, defined as the top 95^th^ percentile of inpatient costs

10. Supplemental Table 6: Sensitivity and specificity for hospital level identification of super-utillizers, defined as the top 95^th^ percentile of inpatient costs

11. Supplemental Figure 6: Rates of chronic medical illness, substance use, and psychiatric use, among super-utilizers defined as the top 95^th^ percentile of inpatient costs.

| **Chronic Comorbidity** | **Beta-Coefficient** | **95% CI** | **p** |
| --- | --- | --- | --- |
| Metastatic Cancer | -0.057 | (-0.072 to -0.042) | <0.001 |
| Lymphoma | -0.056 | (-0.078 to -0.033) | <0.001 |
| Congestive Heart Failure | -0.031 | (-0.04 to -0.022) | <0.001 |
| Chronic Renal Failure | -0.031 | (-0.04 to -0.023) | <0.001 |
| Hypothyroidism | -0.019 | (-0.028 to -0.01) | <0.001 |
| Electrolyte Abnormalities | -0.014 | (-0.022 to -0.005) | 0.001 |
| Rheumatoid arthritis/collagen vascular disease | -0.006 | (-0.021 to 0.009) | 0.464 |
| Acute Blood Loss Anemia | -0.006 | (-0.021 to 0.01) | 0.485 |
| Chronic Lung Disease | -0.002 | (-0.009 to 0.006) | 0.681 |
| Solid tumor without metastasis | -0.001 | (-0.015 to 0.012) | 0.857 |
| Pulmonary Circulation Disorders | 0.004 | (-0.008 to 0.016) | 0.488 |
| Diabetes with chronic complications | 0.008 | (-0.002 to 0.018) | 0.126 |
| Hypertension with and without complications | 0.01 | (0 to 0.02) | 0.044 |
| Coagulation Deficiency | 0.012 | (0.003 to 0.021) | 0.011 |
| Peripheral vascular disease | 0.013 | (0.003 to 0.022) | 0.008 |
| Liver Disease | 0.015 | (0.004 to 0.026) | 0.007 |
| Diabetes without chronic complications | 0.018 | (0.009 to 0.026) | <0.001 |
| Valvular disease | 0.018 | (0.008 to 0.029) | 0.001 |
| Weight Loss | 0.03 | (0.022 to 0.039) | <0.001 |
| HIV and AIDS | 0.034 | (0.002 to 0.067) | 0.038 |
| Deficiency Anemias | 0.036 | (0.028 to 0.044) | <0.001 |
| Depression | 0.04 | (0.033 to 0.048) | <0.001 |
| Other Neurologic Disorders | 0.047 | (0.039 to 0.055) | <0.001 |
| Paralysis | 0.051 | (0.038 to 0.064) | <0.001 |
| Obesity | 0.055 | (0.047 to 0.064) | <0.001 |
| Chronic Peptic Ulcer disease | 0.06 | (-0.008 to 0.128) | 0.086 |
| Alcohol abuse | 0.142 | (0.132 to 0.152) | <0.001 |
| Psychoses | 0.172 | (0.163 to 0.181) | <0.001 |
| Drug Abuse | 0.214 | (0.205 to 0.224) | <0.001 |

**Supplemental Digital Content Table 1:** Independent association between chronic comorbidities and inpatient fragmentation. Association between chronic comorbidity and number of different hospitals admitted to in one year, by multivariate Poisson regression controlling for age, race, gender, insurance status, number of total inpatient encounters in 1 year.

**Supplemental Figure 1:** Distribution of number of encounters stratified by number of hospitals visited in 1 year for super-utilizers (defined as 4 or more admissions in 1 year)

**Supplemental Figure 2:** Distribution of fragmentation with and without inter-hospital transfers treated as a single hospital stay.

|  | **1** | **2** | **3** | **4** | **5** | **6** | **7** | **8** |
| --- | --- | --- | --- | --- | --- | --- | --- | --- |
| **n** | 29687 (40.58%) | 28506 (38.97%) | 10877 (14.87%) | 2874 (3.93%) | 759 (1.04%) | 281 (0.38%) | 118 (0.16%) | 53 (0.07%) |
| **White, n(%)** | 19291 (64.98%) | 17044 (59.79%) | 5994 (55.11%) | 1422 (49.48%) | 347 (45.72%) | 120 (42.7%) | 47 (39.83%) | 21 (39.62%) |
| **Black, n (%)** | 5514 (18.57%) | 5408 (18.97%) | 2093 (19.24%) | 608 (21.16%) | 167 (22%) | 64 (22.78%) | 33 (27.97%) | 16 (30.19%) |
| **Hispanic, n (%)** | 4253 (14.33%) | 5082 (17.83%) | 2329 (21.41%) | 706 (24.57%) | 199 (26.22%) | 77 (27.4%) | 32 (27.12%) | 11 (20.75%) |
| **Other, n (%)** | 629 (2.12%) | 972 (3.41%) | 461 (4.24%) | 138 (4.8%) | 46 (6.06%) | 20 (7.12%) | 6 (5.08%) | 5 (9.43%) |
| **Medicare, (%)** | 67.15% | 64.20% | 59.57% | 52.03% | 48.89% | 40.15% | 35.73% | 41.93% |
| **Medicaid, (%)** | 14.13% | 16.30% | 19.68% | 24.34% | 29.12% | 35.95% | 38.62% | 31.81% |
| **Private (%)** | 10.76% | 10.92% | 11.04% | 9.68% | 8.83% | 8.16% | 6.41% | 4.32% |
| **Selfpay (%)** | 3.65% | 4.24% | 5.63% | 8.91% | 8.92% | 9.93% | 13.15% | 12.36% |
| **Other payer (%)** | 4.30% | 4.34% | 4.07% | 5.04% | 4.23% | 5.81% | 6.08% | 9.59% |
| **Homeless, n (%)** | 244 (0.82%) | 511 (1.79%) | 420 (3.86%) | 233 (8.11%) | 128 (16.86%) | 57 (20.28%) | 31 (26.27%) | 18 (33.96%) |
| **Churn, n (%)** | 1782 (6%) | 2290 (8.03%) | 1214 (11.16%) | 488 (16.98%) | 144 (18.97%) | 59 (21%) | 32 (27.12%) | 21 (39.62%) |
| **Large Metropolitan (>1 million), n (%)** | 17931 (60.4%) | 17572 (61.64%) | 7246 (66.62%) | 2076 (72.23%) | 572 (75.36%) | 217 (77.22%) | 93 (78.81%) | 38 (71.7%) |
| **Small Metropolitan (< 1 million), n (%)** | 9772 (32.92%) | 8443 (29.62%) | 2583 (23.75%) | 532 (18.51%) | 120 (15.81%) | 50 (17.79%) | 14 (11.86%) | 11 (20.75%) |
| **Micropolitan, n (%)** | 1304 (4.39%) | 1697 (5.95%) | 696 (6.4%) | 175 (6.09%) | 44 (5.8%) | 8 (2.85%) | 10 (8.47%) | 3 (5.66%) |
| **Rural, n (%)** | 586 (1.97%) | 751 (2.63%) | 343 (3.15%) | 87 (3.03%) | 22 (2.9%) | 6 (2.14%) | 1 (0.85%) | 1 (1.89%) |
| **Yearly Encounters, mean (SD)** | 4.99 (1.69) | 5.27 (2.01) | 5.89 (2.64) | 7.36 (3.72) | 9.97 (4.83) | 12.56 (5.61) | 15.75 (5.98) | 16.85 (5.99) |
| **Yearly Cost (k), mean (SD)** | 51.84 (46.33) | 56.89 (49.5) | 62.04 (54.48) | 63.33 (52.36) | 73.4 (62.26) | 76.57 (64.43) | 89.45 (76.27) | 95.54 (52.57) |
| **Yearly LOS (days), mean (SD)** | 29.3 (21.36) | 31.32 (23.74) | 35.33 (26.66) | 42.15 (32.46) | 55.44 (39.19) | 62.32 (37.69) | 75.69 (46.68) | 83.04 (42.1) |
| **Psychiatric Disease,n (%)** | 11766 (39.63%) | 13185 (46.25%) | 5966 (54.85%) | 1880 (65.41%) | 576 (75.89%) | 226 (80.43%) | 98 (83.05%) | 44 (83.02%) |
| **Substance Use, n (%)** | 5767 (19.43%) | 7275 (25.52%) | 3905 (35.9%) | 1468 (51.08%) | 508 (66.93%) | 209 (74.38%) | 98 (83.05%) | 48 (90.57%) |
| **Multiple Medical Comorbidities, n (%)** | 26803 (90.29%) | 25633 (89.92%) | 9645 (88.67%) | 2503 (87.09%) | 649 (85.51%) | 249 (88.61%) | 105 (88.98%) | 49 (92.45%) |

**Table IIa:** Patient demographics and yearly costs by state (Florida)

|  | 1 | 2 | 3 | 4 | 5 | 6 | 7 | 8 |
| --- | --- | --- | --- | --- | --- | --- | --- | --- |
| **n** | 9977 (40.67%) | 9072 (36.98%) | 4071 (16.6%) | 1077 (4.39%) | 252 (1.03%) | 52 (0.21%) | 20 (0.08%) | 8 (0.03%) |
| **White, n(%)** | 5541 (55.54%) | 4776 (52.65%) | 1892 (46.48%) | 431 (40.02%) | 96 (38.1%) | 21 (40.38%) | 7 (35%) | 0 (0%) |
| **Black, n (%)** | 3708 (37.17%) | 3465 (38.19%) | 1661 (40.8%) | 472 (43.83%) | 107 (42.46%) | 21 (40.38%) | 8 (40%) | 5 (62.5%) |
| **Hispanic, n (%)** | 387 (3.88%) | 352 (3.88%) | 164 (4.03%) | 54 (5.01%) | 13 (5.16%) | 7 (13.46%) | 1 (5%) | 1 (12.5%) |
| **Other, n (%)** | 341 (3.42%) | 479 (5.28%) | 354 (8.7%) | 120 (11.14%) | 36 (14.29%) | 3 (5.77%) | 4 (20%) | 2 (25%) |
| **Medicare, (%)** | 60.26% | 59.25% | 58.46% | 52.27% | 53.26% | 37.17% | 41.70% | 41.38% |
| **Medicaid, (%)** | 13.90% | 14.99% | 15.97% | 20.19% | 23.46% | 33.26% | 25.00% | 38.04% |
| **Private (%)** | 13.61% | 15.40% | 15.15% | 15.08% | 11.25% | 11.10% | 10.97% | 4.17% |
| **Selfpay (%)** | 6.73% | 5.72% | 5.26% | 7.21% | 7.11% | 13.14% | 20.53% | 11.21% |
| **Other payer (%)** | 5.49% | 4.63% | 5.17% | 5.25% | 4.92% | 5.33% | 1.81% | 5.21% |
| **Homeless, n (%)** | 10 (0.1%) | 16 (0.18%) | 12 (0.29%) | 4 (0.37%) | 1 (0.4%) | 0 (0%) | 0 (0%) | 0 (0%) |
| **Churn, n (%)** | 963 (9.65%) | 952 (10.49%) | 487 (11.96%) | 178 (16.53%) | 51 (20.24%) | 15 (28.85%) | 7 (35%) | 5 (62.5%) |
| **Large Metropolitan (>1 million), n (%)** | 4057 (40.66%) | 4087 (45.05%) | 1917 (47.09%) | 543 (50.42%) | 134 (53.17%) | 25 (48.08%) | 11 (55%) | 6 (75%) |
| **Small Metropolitan (< 1 million), n (%)** | 3699 (37.08%) | 2450 (27.01%) | 912 (22.4%) | 216 (20.06%) | 43 (17.06%) | 10 (19.23%) | 3 (15%) | 2 (25%) |
| **Micropolitan, n (%)** | 1307 (13.1%) | 1260 (13.89%) | 550 (13.51%) | 133 (12.35%) | 31 (12.3%) | 10 (19.23%) | 1 (5%) | 0 (0%) |
| **Rural, n (%)** | 906 (9.08%) | 1275 (14.05%) | 692 (17%) | 185 (17.18%) | 44 (17.46%) | 7 (13.46%) | 5 (25%) | 0 (0%) |
| **Yearly Encounters, mean (SD)** | 4.94 (1.75) | 5.14 (1.79) | 5.51 (2.17) | 6.42 (2.93) | 8.36 (3.59) | 10.02 (4.15) | 12.4 (3.55) | 14.25 (4.17) |
| **Yearly Cost (k), mean (SD)** | 183.55 (160.24) | 223.29 (209.82) | 267.23 (262.65) | 328.74 (327.16) | 421.96 (471.34) | 397.84 (420.42) | 271.14 (164.26) | 389.98 (299.88) |
| **Yearly LOS (days), mean (SD)** | 28.98 (19.76) | 34.03 (25.07) | 41.78 (31.56) | 52.26 (38.51) | 66.69 (45.48) | 68.12 (49.42) | 63.6 (34.68) | 81.63 (48.92) |
| **Psychiatric Disease,n (%)** | 3587 (35.95%) | 3737 (41.19%) | 2043 (50.18%) | 645 (59.89%) | 168 (66.67%) | 35 (67.31%) | 16 (80%) | 4 (50%) |
| **Substance Use, n (%)** | 1702 (17.06%) | 1940 (21.38%) | 1156 (28.4%) | 448 (41.6%) | 124 (49.21%) | 32 (61.54%) | 14 (70%) | 6 (75%) |
| **Multiple Medical Comorbidities, n (%)** | 8944 (89.65%) | 8326 (91.78%) | 3711 (91.16%) | 975 (90.53%) | 236 (93.65%) | 49 (94.23%) | 17 (85%) | 7 (87.5%) |

**Table IIb:** Patient demographics and yearly costs by state (Georgia)

|  | 1 | 2 | 3 | 4 | 5 | 6 |
| --- | --- | --- | --- | --- | --- | --- |
| **n** | 3105 (0.53%) | 2217 (0.38%) | 501 (0.09%) | 57 (0.01%) | 4 (0%) | 1 (0%) |
| **White, n(%)** | 2856 (91.98%) | 2032 (91.66%) | 457 (91.22%) | 51 (89.47%) | 3 (75%) | 1 (100%) |
| **Black, n (%)** | 137 (4.41%) | 113 (5.1%) | 28 (5.59%) | 3 (5.26%) | 0 (0%) | 0 (0%) |
| **Hispanic, n (%)** | 40 (1.29%) | 40 (1.8%) | 6 (1.2%) | 3 (5.26%) | 1 (25%) | 0 (0%) |
| **Other, n (%)** | 72 (2.32%) | 32 (1.44%) | 10 (2%) | 0 (0%) | 0 (0%) | 0 (0%) |
| **Medicare, (%)** | 74.14% | 69.30% | 64.22% | 59.02% | 42.86% | 100.00% |
| **Medicaid, (%)** | 8.18% | 10.69% | 16.56% | 17.01% | 23.57% | 0.00% |
| **Private (%)** | 15.34% | 15.70% | 14.00% | 9.47% | 23.33% | 0.00% |
| **Selfpay (%)** | 1.58% | 3.17% | 3.76% | 11.11% | 5.24% | 0.00% |
| **Other payer (%)** | 0.75% | 1.14% | 1.46% | 3.39% | 5.00% | 0.00% |
| **Homeless, n (%)** | 0 (0%) | 0 (0%) | 0 (0%) | 0 (0%) | 0 (0%) | 0 (%) |
| **Churn, n (%)** | 106 (3.41%) | 159 (7.17%) | 52 (10.38%) | 16 (28.07%) | 2 (50%) | 0 (%) |
| **Large Metropolitan (>1 million), n (%)** | 0 (0%) | 0 (0%) | 0 (0%) | 0 (0%) | 0 (0%) | 0 (0%) |
| **Small Metropolitan (< 1 million), n (%)** | 1881 (60.58%) | 1050 (47.36%) | 183 (36.53%) | 22 (38.6%) | 3 (75%) | 0 (0%) |
| **Micropolitan, n (%)** | 489 (15.75%) | 446 (20.12%) | 119 (23.75%) | 9 (15.79%) | 1 (25%) | 0 (0%) |
| **Rural, n (%)** | 735 (23.67%) | 721 (32.52%) | 199 (39.72%) | 26 (45.61%) | 0 (0%) | 1 (100%) |
| **Yearly Encounters, mean (SD)** | 4.85 (1.45) | 5.04 (1.73) | 5.48 (2.1) | 6.58 (2.69) | 8.75 (4.35) | 8 (4.35) |
| **Yearly Cost (k), mean (SD)** | 53.21 (44.65) | 58.11 (43.44) | 64.43 (48.9) | 69.83 (51.26) | 70.75 (34.95) | 73.94 (34.95) |
| **Yearly LOS (days), mean (SD)** | 25.74 (17.19) | 26.39 (18.06) | 28.03 (18.22) | 34.65 (27.77) | 32.25 (18.52) | 16 (18.52) |
| **Psychiatric Disease,n (%)** | 1361 (43.83%) | 1014 (45.74%) | 254 (50.7%) | 34 (59.65%) | 3 (75%) | 1 (100%) |
| **Substance Use, n (%)** | 339 (10.92%) | 326 (14.7%) | 90 (17.96%) | 20 (35.09%) | 3 (75%) | 1 (100%) |
| **Multiple Medical Comorbidities, n (%)** | 2805 (90.34%) | 2048 (92.38%) | 458 (91.42%) | 55 (96.49%) | 4 (100%) | 1 (100%) |

**Table IIc:** Patient demographics and yearly costs by state (Iowa)

|  | 1 | 2 | 3 | 4 | 5 | 6 | 7 | 8 |
| --- | --- | --- | --- | --- | --- | --- | --- | --- |
| **n** | 1383 (44.84%) | 1126 (36.51%) | 421 (13.65%) | 116 (3.76%) | 30 (0.97%) | 5 (0.16%) | 2 (0.06%) | 1 (0.03%) |
| **White, n(%)** | 443 (32.03%) | 505 (44.85%) | 213 (50.59%) | 51 (43.97%) | 14 (46.67%) | 0 (0%) | 0 (0%) | 0 (0%) |
| **Black, n (%)** | 11 (0.8%) | 11 (0.98%) | 6 (1.43%) | 0 (0%) | 1 (3.33%) | 1 (20%) | 0 (0%) | 0 (0%) |
| **Hispanic, n (%)** | 75 (5.42%) | 132 (11.72%) | 76 (18.05%) | 26 (22.41%) | 9 (30%) | 2 (40%) | 0 (0%) | 0 (0%) |
| **Other, n (%)** | 854 (61.75%) | 478 (42.45%) | 126 (29.93%) | 39 (33.62%) | 6 (20%) | 2 (40%) | 2 (100%) | 1 (100%) |
| **Medicare, (%)** | 52.41% | 54.26% | 43.84% | 33.93% | 33.24% | 20.00% | 50.00% | 0.00% |
| **Medicaid, (%)** | 12.29% | 13.61% | 18.60% | 25.30% | 32.49% | 46.33% | 50.00% | 0.00% |
| **Private (%)** | 28.25% | 24.44% | 25.76% | 21.18% | 15.18% | 13.67% | 0.00% | 100.00% |
| **Selfpay (%)** | 2.93% | 3.62% | 8.04% | 10.71% | 12.58% | 17.78% | 0.00% | 0.00% |
| **Other payer (%)** | 4.12% | 4.06% | 3.75% | 8.89% | 6.50% | 2.22% | 0.00% | 0.00% |
| **Homeless, n (%)** | 0 (0%) | 0 (0%) | 0 (0%) | 1 (0.86%) | 0 (0%) | 244 (0.82%) | 0 (0%) | 0 (0%) |
| **Churn, n (%)** | 69 (4.99%) | 90 (7.99%) | 72 (17.1%) | 30 (25.86%) | 11 (36.67%) | 1782 (6%) | 0 (0%) | 0 (0%) |
| **Large Metropolitan (>1 million), n (%)** | 11 (0.8%) | 3 (0.27%) | 1 (0.24%) | 0 (0%) | 0 (0%) | 0 (0%) | 0 (0%) | 0 (0%) |
| **Small Metropolitan (< 1 million), n (%)** | 1224 (88.5%) | 940 (83.48%) | 358 (85.04%) | 103 (88.79%) | 28 (93.33%) | 5 (100%) | 2 (100%) | 1 (100%) |
| **Micropolitan, n (%)** | 87 (6.29%) | 102 (9.06%) | 20 (4.75%) | 7 (6.03%) | 0 (0%) | 0 (0%) | 0 (0%) | 0 (0%) |
| **Rural, n (%)** | 61 (4.41%) | 81 (7.19%) | 42 (9.98%) | 6 (5.17%) | 2 (6.67%) | 0 (0%) | 0 (0%) | 0 (0%) |
| **Yearly Encounters, mean (SD)** | 4.86 (1.43) | 4.98 (1.59) | 5.21 (1.88) | 6.51 (2.71) | 7.83 (1.88) | 10.6 (3.05) | 15 (2.83) |  |
| **Yearly Cost (k), mean (SD)** | 73.14 (78.66) | 61.98 (50.05) | 56.93 (61.38) | 53.75 (37.95) | 73.29 (44.3) | 62.86 (21.37) | 78.03 (30.08) |  |
| **Yearly LOS (days), mean (SD)** | 26.18 (20.66) | 23.9 (15.91) | 25.94 (17.51) | 31.06 (21.02) | 42.1 (30.02) | 43 (9.22) | 64.5 (33.23) |  |
| **Psychiatric Disease,n (%)** | 463 (33.48%) | 444 (39.43%) | 224 (53.21%) | 84 (72.41%) | 26 (86.67%) | 4 (80%) | 2 (100%) | 0 (0%) |
| **Substance Use, n (%)** | 207 (14.97%) | 258 (22.91%) | 180 (42.76%) | 71 (61.21%) | 21 (70%) | 3 (60%) | 2 (100%) | 1 (100%) |
| **Multiple Medical Comorbidities, n (%)** | 1075 (77.73%) | 894 (79.4%) | 290 (68.88%) | 81 (69.83%) | 23 (76.67%) | 4 (80%) | 2 (100%) | 0 (0%) |

**Table IId:** Patient demographics and yearly costs by state (Utah)

|  | 1 | 2 | 3 | 4 | 5 | 6 | 7 | 8 |
| --- | --- | --- | --- | --- | --- | --- | --- | --- |
| **n** | 25394 (42.29%) | 22085 (36.78%) | 8637 (14.38%) | 2489 (4.15%) | 767 (1.28%) | 363 (0.6%) | 207 (0.34%) | 100 (0.17%) |
| **White, n(%)** | 13488 (53.11%) | 10404 (47.11%) | 3208 (37.14%) | 664 (26.68%) | 143 (18.64%) | 52 (14.33%) | 22 (10.63%) | 7 (0.07%) |
| **Black, n (%)** | 4709 (18.54%) | 3924 (17.77%) | 1713 (19.83%) | 563 (22.62%) | 200 (26.08%) | 90 (24.79%) | 51 (24.64%) | 28 (0.28%) |
| **Hispanic, n (%)** | 3400 (13.39%) | 2431 (11.01%) | 990 (11.46%) | 295 (11.85%) | 89 (11.6%) | 45 (12.4%) | 27 (13.04%) | 10 (0.1%) |
| **Other, n (%)** | 3797 (14.95%) | 5326 (24.12%) | 2726 (31.56%) | 967 (38.85%) | 335 (43.68%) | 176 (48.48%) | 107 (51.69%) | 55 (0.55%) |
| **Medicare, (%)** | 62.16% | 58.79% | 51.83% | 37.33% | 29.29% | 21.16% | 20.46% | 19.69% |
| **Medicaid, (%)** | 19.96% | 23.10% | 32.15% | 48.84% | 61.02% | 68.08% | 72.19% | 73.68% |
| **Private (%)** | 14.89% | 14.73% | 12.28% | 9.08% | 5.49% | 5.08% | 3.70% | 2.12% |
| **Selfpay (%)** | 1.34% | 1.61% | 1.91% | 2.63% | 2.30% | 3.27% | 2.45% | 2.70% |
| **Other payer (%)** | 1.66% | 1.76% | 1.83% | 2.12% | 1.90% | 2.40% | 1.20% | 1.81% |
| **Homeless, n (%)** | 1057 (4.16%) | 2316 (10.49%) | 1805 (20.9%) | 972 (39.05%) | 424 (55.28%) | 240 (66.12%) | 161 (77.78%) | 84 (84%) |
| **Churn, n (%)** | 781 (3.08%) | 958 (4.34%) | 494 (5.72%) | 236 (9.48%) | 87 (11.34%) | 59 (16.25%) | 34 (16.43%) | 17 (17%) |
| **Large Metropolitan (>1 million), n (%)** | 19252 (75.81%) | 16841 (76.26%) | 6938 (80.33%) | 2084 (83.73%) | 670 (87.35%) | 317 (87.33%) | 183 (88.41%) | 86 (86%) |
| **Small Metropolitan (< 1 million), n (%)** | 4283 (16.87%) | 3428 (15.52%) | 1054 (12.2%) | 261 (10.49%) | 71 (9.26%) | 32 (8.82%) | 22 (10.63%) | 13 (13%) |
| **Micropolitan, n (%)** | 1410 (5.55%) | 1278 (5.79%) | 408 (4.72%) | 79 (3.17%) | 16 (2.09%) | 10 (2.75%) | 1 (0.48%) | 0 (0%) |
| **Rural, n (%)** | 425 (1.67%) | 535 (2.42%) | 237 (2.74%) | 65 (2.61%) | 10 (1.3%) | 4 (1.1%) | 1 (0.48%) | 1 (1%) |
| **Yearly Encounters, mean (SD)** | 4.96 (1.61) | 5.17 (1.86) | 5.69 (2.39) | 6.91 (3.19) | 9.31 (4.54) | 11.32 (4.9) | 13.87 (5.72) | 15.7 (5.5) |
| **Yearly Cost (k), mean (SD)** | 71.3 (65.36) | 76.74 (67.03) | 78.81 (70.36) | 76.32 (67.76) | 85.05 (73.17) | 90.87 (65.98) | 97.5 (65.86) | 113.39 (78.61) |
| **Yearly LOS (days), mean (SD)** | 34.67 (26.7) | 37.69 (29.77) | 42.42 (33.98) | 49.92 (38.99) | 60.64 (44.13) | 70.81 (46.53) | 78.13 (48.41) | 88.78 (55.35) |
| **Psychiatric Disease,n (%)** | 9108 (35.87%) | 9240 (41.84%) | 4322 (50.04%) | 1593 (64%) | 553 (72.1%) | 273 (75.21%) | 163 (78.74%) | 77 (77%) |
| **Substance Use, n (%)** | 4188 (16.49%) | 5216 (23.62%) | 3182 (36.84%) | 1429 (57.41%) | 566 (73.79%) | 290 (79.89%) | 170 (82.13%) | 81 (81%) |
| **Multiple Medical Comorbidities, n (%)** | 22025 (86.73%) | 18990 (85.99%) | 6947 (80.43%) | 1747 (70.19%) | 553 (72.1%) | 268 (73.83%) | 149 (71.98%) | 77 (77%) |

**Table IId:** Patient demographics and yearly costs by state (New York)

|  | 1 | 2 | 3 | 4 |
| --- | --- | --- | --- | --- |
| **n** | 565 (68.9%) | 233 (28.41%) | 20 (2.44%) | 2 (0.24%) |
| **White, n(%)** | 548 (96.99%) | 228 (97.85%) | 18 (90%) | 2 (100%) |
| **Black, n (%)** | 4 (0.71%) | 1 (0.43%) | 1 (5%) | 0 (0%) |
| **Hispanic, n (%)** | 0 (0%) | 0 (0%) | 0 (0%) | 0 (0%) |
| **Other, n (%)** | 13 (2.3%) | 4 (1.72%) | 1 (5%) | 0 (0%) |
| **Medicare, (%)** | 65.59% | 62.67% | 60.00% | 0.00% |
| **Medicaid, (%)** | 14.62% | 19.64% | 33.00% | 100.00% |
| **Private (%)** | 14.60% | 12.53% | 5.00% | 0.00% |
| **Selfpay (%)** | 0.27% | 1.17% | 0.00% | 0.00% |
| **Other payer (%)** | 4.92% | 3.99% | 2.00% | 0.00% |
| **Homeless, n (%)** | 0 (0%) | 0 (0%) | 0 (0%) | 0 (%) |
| **Churn, n (%)** | 6 (1.06%) | 8 (3.43%) | 0 (0%) | 0 (%) |
| **Large Metropolitan (>1 million), n (%)** | 1 (0.18%) | 0 (0%) | 0 (0%) | 0 (0%) |
| **Small Metropolitan (< 1 million), n (%)** | 211 (37.35%) | 52 (22.32%) | 1 (5%) | 0 (0%) |
| **Micropolitan, n (%)** | 273 (48.32%) | 106 (45.49%) | 10 (50%) | 0 (0%) |
| **Rural, n (%)** | 80 (14.16%) | 75 (32.19%) | 9 (45%) | 2 (100%) |
| **Yearly Encounters, mean (SD)** | 4.92 (1.55) | 5.02 (1.48) | 5.55 (1.76) | 8 (5.66) |
| **Yearly Cost (k), mean (SD)** | 67.29 (47.36) | 75.15 (49.93) | 78.31 (57.67) | 41.66 (31.11) |
| **Yearly LOS (days), mean (SD)** | 25.78 (19.68) | 26.72 (21.7) | 31.85 (26.76) | 19 (1.41) |
| **Psychiatric Disease,n (%)** | 298 (52.74%) | 114 (48.93%) | 14 (70%) | 2 (100%) |
| **Substance Use, n (%)** | 118 (20.88%) | 52 (22.32%) | 9 (45%) | 1 (50%) |
| **Multiple Medical Comorbidities, n (%)** | 502 (88.85%) | 206 (88.41%) | 15 (75%) | 2 (100%) |

**Table IIf:** Patient demographics and yearly costs by state (Vermont)**Supplemental Digital Content Figure 3:** Prevalence rate of different categories of chronic conditions stratified by the number of hospitals admitted to in a 1 year period for super utilizers defined as the top 95^th^ percentile of inpatient costs.

|  | One Hospital | | 2 or More Hospitals | | Total | |
| --- | --- | --- | --- | --- | --- | --- |
|  | Sensitivity | Specificity | Sensitivity | Specificity | Sensitivity | Specificity |
| 3 or more encounters | 100% | 96.81% | 51.73% | 100% | 85.23% | 98.03% |
| 4 or more encounters | 100% | 100% | 36.10% | 100% | 62.85% | 100% |
| Length of Stay > 95 percentile | 81.54% | 93.51% | 64.00% | 83.34% | 71.34% | 92.81% |
| Yearly Cost > 95 percentile | 63.71% | 93.92% | 47.52% | 85.96% | 54.30% | 93.37% |

**Table III: Sensitivity and specificity of measures to identify super utilizers from individual hospitals; Super-utilizers defined as 4 or more hospitals in a 1 year period.**

|  | 1 | 2 | 3 | 4 | 5 | 6 | 7 | 8 |
| --- | --- | --- | --- | --- | --- | --- | --- | --- |
| **n** | 112528 (59.43%) | 56912 (30.05%) | 14753 (7.79%) | 3342 (1.76%) | 1047 (0.55%) | 431 (0.23%) | 225 (0.12%) | 122 (0.06%) |
| **White, n(%)** | 67786 (60.24%) | 31636 (55.59%) | 6883 (46.65%) | 1290 (38.6%) | 350 (33.43%) | 119 (27.61%) | 52 (23.11%) | 20 (16.39%) |
| **Black, n (%)** | 19978 (17.75%) | 10852 (19.07%) | 3420 (23.18%) | 885 (26.48%) | 282 (26.93%) | 110 (25.52%) | 62 (27.56%) | 41 (33.61%) |
| **Hispanic, n (%)** | 12251 (10.89%) | 6891 (12.11%) | 2137 (14.49%) | 550 (16.46%) | 176 (16.81%) | 85 (19.72%) | 40 (17.78%) | 15 (12.3%) |
| **Other, n (%)** | 12513 (11.12%) | 7533 (13.24%) | 2313 (15.68%) | 617 (18.46%) | 239 (22.83%) | 117 (27.15%) | 71 (31.56%) | 46 (37.7%) |
| **Medicare, (%)** | 56.70% | 63.16% | 62.71% | 55.06% | 47.25% | 34.04% | 30.71% | 30.60% |
| **Medicaid, (%)** | 13.87% | 14.30% | 18.39% | 26.78% | 37.73% | 49.76% | 54.73% | 56.42% |
| **Private (%)** | 22.03% | 16.59% | 13.30% | 10.62% | 7.42% | 6.15% | 4.15% | 3.34% |
| **Selfpay (%)** | 3.21% | 2.69% | 2.69% | 3.80% | 4.35% | 5.76% | 7.73% | 5.72% |
| **Other payer (%)** | 4.19% | 3.26% | 2.92% | 3.74% | 3.26% | 4.29% | 2.69% | 3.91% |
| **Homeless, n (%)** | 4645 (4.13%) | 2925 (5.14%) | 992 (6.72%) | 347 (10.38%) | 143 (13.66%) | 69 (16.01%) | 41 (18.22%) | 31 (25.41%) |
| **Churn, n (%)** | 1368 (1.22%) | 1815 (3.19%) | 937 (6.35%) | 462 (13.82%) | 248 (23.69%) | 168 (38.98%) | 110 (48.89%) | 72 (59.02%) |
| **Large Metropolitan (>1 million), n (%)** | 69788 (62.02%) | 35478 (62.34%) | 9708 (65.8%) | 2328 (69.66%) | 772 (73.73%) | 345 (80.05%) | 185 (82.22%) | 98 (80.33%) |
| **Small Metropolitan (< 1 million), n (%)** | 30063 (26.72%) | 13961 (24.53%) | 2978 (20.19%) | 580 (17.35%) | 159 (15.19%) | 60 (13.92%) | 27 (12%) | 21 (17.21%) |
| **Micropolitan, n (%)** | 7110 (6.32%) | 4287 (7.53%) | 1130 (7.66%) | 226 (6.76%) | 56 (5.35%) | 14 (3.25%) | 8 (3.56%) | 2 (1.64%) |
| **Rural, n (%)** | 5236 (4.65%) | 3156 (5.55%) | 934 (6.33%) | 208 (6.22%) | 60 (5.73%) | 12 (2.78%) | 5 (2.22%) | 1 (0.82%) |
| **Yearly Encounters, mean (SD)** | 2.68 (2.08) | 4.18 (2.4) | 5.83 (3.02) | 8.06 (4.01) | 10.62 (5.17) | 13.01 (5.79) | 16.04 (6.08) | 16.98 (5.92) |
| **Yearly Cost (k), mean (SD)** | 126.57 (126.7) | 140.65 (152.97) | 166.28 (187.88) | 179.8 (219.39) | 189.79 (273.09) | 148.13 (183.61) | 136.04 (93.64) | 142.99 (119.77) |
| **Yearly LOS (days), mean (SD)** | 34.58 (29.51) | 41.38 (30.05) | 51.87 (34.49) | 64.06 (40.53) | 76.81 (45.68) | 84.76 (44.89) | 94.79 (47.49) | 98.89 (51.37) |
| **Psychiatric Disease,n (%)** | 26445 (23.5%) | 19364 (34.02%) | 6881 (46.64%) | 2044 (61.16%) | 766 (73.16%) | 344 (79.81%) | 184 (81.78%) | 92 (75.41%) |
| **Substance Use, n (%)** | 13451 (11.95%) | 9570 (16.82%) | 3862 (26.18%) | 1475 (44.14%) | 664 (63.42%) | 329 (76.33%) | 188 (83.56%) | 107 (87.7%) |
| **Multiple Medical Comorbidities, n (%)** | 87599 (77.85%) | 52105 (91.55%) | 13983 (94.78%) | 3122 (93.42%) | 963 (91.98%) | 386 (89.56%) | 199 (88.44%) | 105 (86.07%) |

**Table 4:** Patient demographics and yearly costs stratified by the number of hospitals admitted to in 1 year for super-utilizers; defined as top 95 percentile of yearly costs per state)

**Supplemental Figure 4:** Distribution of number of encounters per year by fragmentation of super-utilizers defined as the top 95^th^ percentile of inpatient costs.

**Supplemental Figure 5:** Comparing 4 different single hospital methods to detect super-utilizers: 3 and 4 or more inpatient encounters, and top 5th percentile for either inpatient days or inpatient cost. We determined the likelihood that a single hospital would correctly identify a patient as a super-utilizer as defined as the top 95^th^ percentile of yearly inpatient costs per state.

|  | One Hospital | 2 or More Hospitals | Total |
| --- | --- | --- | --- |
| 3 or more encounters | 0.681 (0.681 to 0.684) | 0.677 (0.675 to 0.679) | 0.696 (0.694 to 0.698) |
| 4 or more encounters | 0.617 (0.617 yo 0.619) | 0.623 (0.620 to 0.622) | 0.625 (0.625 to 0.627) |
| Length of Stay > 95 percentile | 0.863 (0.862 to 0.865) | 0.813 (0.866 to 0.815) | 0.860 (0.859 to 0.861) |
| Yearly Cost > 95 percentile | 0.882 (0.881 to 0.883) | 0.816 (0.814 to 0.818) | 0.863 (0.862 to 0.864) |

**Supplemental Table 5: : Individual hospitals ability to accurately capture super-utilizers as identified as the top 95^th^ percentile for inpatient cost** C-statistic with 95% Confidence interval based on an individual patient’s maximum use at a single hospital.

**Supplemental Figure 6:** Prevalence rate of different categories of chronic conditions stratified by the number of hospitals admitted to in a 1 year period for super utilizers defined as the top 95^th^ percentile of inpatient costs.

|  | One Hospital | | 2 or More Hospitals | | Total | |
| --- | --- | --- | --- | --- | --- | --- |
|  | Sensitivity | Specificity | Sensitivity | Specificity | Sensitivity | Specificity |
| 3 or more encounters | 41% | 96.03% | 48.60% | 87% | 43.82% | 95.35% |
| 4 or more encounters | 25% | 99% | 29.17% | 95.28% | 26.66% | 98% |
| Length of Stay > 95 percentile | 78.35% | 94.36% | 78.75% | 84% | 78.51% | 93.58% |
| Yearly Cost > 95 percentile | 81.14% | 95.24% | 72.63% | 90.55% | 78.30% | 93.32% |

**Table 6: Sensitivity and specificity of measures to identify super utilizers from individual hospitals; Super-utilizers defined as top 95 percentile of inpatient costs.**
